# Occurrence of Hypoxemia the First Day After Trauma Assessed by Continuous Pulse Oximetry

**DOI:** 10.1111/aas.70220

**Published:** 2026-03-16

**Authors:** Jacob Jensen‐Abbew, Emma Atsuko Tsuchiya, Tobias Arleth, Felicia Dinesen, Carl Johan Queitsch, Martin Von Magius, Michelle Icka Christensen, Karl Peter Damgård Madsen, Oscar Rosenkrantz, Jacob Steinmetz

**Affiliations:** ^1^ Department of Anaesthesiology, Centre of Head and Orthopaedics Copenhagen University Hospital—Rigshospitalet Copenhagen Denmark; ^2^ Department of Clinical Epidemiology Aarhus University Hospital and Aarhus University Aarhus Denmark; ^3^ Norwegian Air Ambulance Foundation Oslo Norway; ^4^ Danish Air Ambulance Aarhus Denmark; ^5^ Faculty of Health Aarhus University Aarhus Denmark

## Abstract

**Background:**

Trauma is a leading cause of death and disability in young adults. Although supplemental oxygen is recommended early after trauma to prevent hypoxemia, evidence regarding the occurrence and distribution of hypoxemic episodes, to raise awareness on potential clinical implications, is sparse. The aim of this study was to investigate the occurrence and daily distribution of hypoxemia within the first day of admission after trauma using continuous pulse oximetry.

**Methods:**

Adult trauma patients admitted through the trauma centre at Rigshospitalet, Denmark, between February 20 and August 24, 2024, were included in this observational study irrespective of subsequent admission department. Arterial oxygen saturation measured by pulse oximetry (SpO_2_) was continuously monitored for 24 h to identify clinically relevant hypoxemic episodes, defined as SpO_2_ < 90%, for > 5 min. The incidence of episodes was compared regarding the occurrence between daytime and nighttime. Hypoxemic episodes were hypothesized to be more frequent during nighttime.

**Results:**

Among 165 included participants, data from 155 were analyzed. Median age was 49 years (IQR 31–62), 74.8% were male, and median Injury Severity Score was 13 (IQR 9–19). In total 146 episodes were recorded, and both daytime and nighttime periods showed incidence rates of 5.1 episodes per 100 participant‐hours, yielding an incidence rate ratio (IRR) of 1.01 (95% CI, 0.73–1.4; *p* = 0.95) between daytime and nighttime. No differences between daytime and nighttime were found in cumulative hypoxemia duration, prolonged hypoxemic episodes, or across hospital locations.

**Conclusion:**

On average, 5.1 clinically relevant hypoxemic episodes occurred per 100 participant‐hours of continuous SpO_2_ monitoring during the first 24 h of hospitalization following trauma. The study found similar incidence rates of clinically relevant hypoxemic episodes at daytime and nighttime.

**Editorial Comment:**

Supplementary oxygen is recommended in the early phase after trauma to prevent hypoxaemia. This single center study confirmed that clinically relevant hypoxaemic episodes occur on average five times per 100 participant‐hours during the first 24 h of hospitalization following trauma, with similar incidences during both day and night. The study highligths the importance of continuous monitoring of peripheral oxygenation including if trauma patients are transferred from high‐depency units to the wards.

**Trial Registration:** ClinicalTrials.gov: NCT06256692

**Abbreviations**
bpm: beats per minute
CI: confidence interval
ICU: intensive care unit
IQR: interquartile range
IR: incidence rate
IRR: incidence rate ratio
MRI: magnetic resonance imaging
SpO_2_
: arterial oxygen saturation measured by continuous pulse oximetry

## 1 Introduction

### 1.1 Background

Trauma is a leading cause of death and disability worldwide, the primary cause of disability‐adjusted life years in individuals younger than 50 years, and the leading cause of death among adults younger than 45 years in the Western world [1, 2]. The Advanced Trauma Life Support (ATLS) guidelines highlight the serious risks of adverse outcomes associated with hypoxemia following trauma [3]. Consequently, supplemental oxygen is recommended for all severely injured trauma patients during the initial phase after trauma to prevent hypoxemia, though the duration of this phase is not explicitly defined. This recommendation lacks unequivocal evidence, and studies have reported inconsistent decision‐making regarding supplemental oxygen in the initial phase, including administration without clear indications [4, 5, 6].

Varying incidences of hypoxemia have been reported in both prehospital and in‐hospital trauma studies [6, 7, 8, 9, 10]. Additionally, hypoxemia has been associated with increased adverse outcomes and in‐hospital mortality [8, 11, 12, 13, 14].

In surgical patients, arterial oxygen saturation measured by continuous pulse oximetry (SpO_2_) appears more effective in detecting hypoxemia than when measured in intervals, for example, hourly [15, 16, 17, 18], and early implementation has been recommended for all trauma patients [19]. Studies on healthy adults, surgical patients, and cardiac patients with myocardial infarction have shown a tendency for hypoxemia to accumulate during nighttime [20, 21, 22]. This finding raises concerns that a similar pattern may exist among trauma patients, with potential clinical implications for patient outcomes.

To our knowledge, the occurrence and daily distribution of in‐hospital hypoxemia under standard post‐trauma care remain largely uninvestigated, and it remains unclear to what extent hypoxemia occurs within the first 24 h of hospitalization following trauma.

### 1.2 Goals of Investigation

This study aimed to investigate the occurrence and daily distribution of hypoxemia within the first 24 h of hospitalization, irrespective of subsequent admission department, following trauma using continuous SpO_2_ measurements. The hypothesis was that hypoxemic episodes would occur more frequently at nighttime (20:00–07:59) than during daytime (08:00–19:59).

## 2 Patients and Methods

### 2.1 Study Design and Setting

This study was a single center, observational study of trauma patients at Rigshospitalet, Copenhagen University Hospital, Denmark, the only major trauma center in the eastern part of Denmark with 2.7 million residents, treating approximately 1000 trauma patients annually [23].

Admission to the trauma centre at Rigshospitalet is based on predefined criteria related to mechanism of injury, physiological or anatomical considerations.

### 2.2 Ethics Committee Approval

The study was approved (H‐23065206) on January 3, 2024, with a supplemental approval (110381) on April 4, 2024, by the Regional Research Ethics Committee in the Capital Region, Denmark.

### 2.3 Selection of Participants

All trauma patients admitted to the trauma centre at Rigshospitalet during the study period were screened for inclusion, with formal inclusion determined by the trauma team leader. Initially, two pulse oximeters were used for data collection, and a further two were added in mid‐May 2024.

Inclusion criteria were trauma team activation, age ≥ 18 years, blunt or penetrating trauma, and hospital admission after initial resuscitation in the trauma center to a ward, intensive care unit (ICU), or directly to an operating room. Exclusion criteria included suspected carbon monoxide intoxication, no suitable finger or toe for sensor attachment, cancelation of trauma team activation before arrival, or pulse oximeter unavailability.

### 2.4 Participant Consent

Informed consent was obtained for all participants in accordance with the Danish Medical Research Ethics Committee's emergency research procedure. For participants unable to consent, proxy consent was obtained from the next‐of‐kin and an independent clinician, remaining valid if participant consent was unobtainable within 30 days. Consent from the independent clinician stood alone if the next‐of‐kin could not be identified or contacted after multiple attempts.

### 2.5 Measurements

Continuous pulse oximetry was used to measure SpO_2_ with the Nellcor Portable SpO_2_ Patient Monitoring System, PM10N (Medtronic, Minneapolis, USA), placed on the participants´ index finger or alternatively on another finger or toe if necessary. The Nellcor Flexible SpO_2_ Reusable Sensor was primarily used, in a few cases the Nellcor adhesive SpO_2_ sensor was used instead. Recording was continuous (every second), with the use of project specific equipment in addition to the clinically used standard monitoring, aiming for 24 uninterrupted hours. However, premature probe removal would shut down the device, necessitating a restart to continue recording, resulting in missing data for the duration the device remained off. The pulse oximeter was removed after 24 h, upon discharge or hospital transfer, which ever came first. A measurement was defined as a one‐second reading within the designated day‐ or nighttime period.

To minimize interference with standard care, alarms were set restrictively and would activate if SpO_2_ < 80%, pulse rate < 40 beats per minute (bpm) or > 140 bpm. Clinical responses to potential alarms were not standardized and were left to the discretion of healthcare personnel; intervention data were not systematically collected.

### 2.6 Data and Variables

In addition to measured SpO_2_ data, Injury Severity Scores and Abbreviated Injury Scale codes were obtained from the Danish Trauma Registry, where certified personnel perform Abbreviated Injury Scale coding and Injury Severity Scores calculations. Participant characteristics, as well as prehospital, trauma center, and admission conditions (including drug administration) were extracted from participants medical records.

Drugs were classified as potentially respiratory depressing based on their pharmacological effects and documented side effects (Table S1).

### 2.7 Outcomes

The primary outcome of the study was the occurrence and distribution of clinically relevant hypoxemic episodes, assessed as incidence rates (IRs) of episodes during daytime (08:00–19:59) and nighttime (20:00–07:59) within the first 24 h of hospitalization following trauma. No universally accepted definition of hypoxemic episodes exists. In this study we defined a clinically relevant hypoxemic episode as SpO_2_ < 90%, for > 5 min.

Secondary outcomes included the IRs of hypoxemic episodes across different hospital locations, including the trauma center, ICU, operating room, recovery room, and ward. Additionally, we examined the IRs of prolonged hypoxemic episodes (SpO_2_ < 90%, for > 30 min) and the cumulative duration of hypoxemia (total time with SpO_2_ < 90%).

The sample size calculation was based on the number of participants experiencing hypoxemic episodes. After data collection we changed the primary outcome definition to IRs of hypoxemic episodes. The change was made prior to final analysis because simple counts were misleading given widely varying at‐risk time (time under monitoring), ranging from 29 min to 24 h, which IRs appropriately account for. Furthermore, we considered IRs of greater clinical relevance and necessary to allow for meaningful comparisons across hospital locations with differing average at‐risk times. All protocolized outcomes and analyses were reported.

### 2.8 Statistics and Analysis

To account for artifacts during measuring, we applied the method described by Haahr‐Raunkjær et al. which defined artifacts as SpO_2_ values following a change of more than 4% per second [24]. When such episodes occurred, the subsequent SpO_2_ value was replaced with a designated missing value code. Furthermore, when identifying episodes of hypoxemia, up to 10 consecutive seconds of missing SpO_2_ values without terminating the episode were allowed.

Ideally, all participants would have complete 24‐h monitoring. However, the actual duration sometimes varied, why participants could have SpO_2_ measurements during either daytime, nighttime, or both. Hence, the data were treated as unpaired and analyzed as independent groups, with all results stratified accordingly.

Continuous variables were presented as medians with interquartile ranges (IQRs), while categorical variables were reported as counts and percentages.

Poisson regression was used to calculate incidence rate ratios (IRRs), with 95% confidence intervals (CIs) and *p* values estimated using the Wald method when the number of episodes in both the daytime and nighttime groups exceeded 5. For comparisons with fewer events, the exact Poisson test was used. The Mann–Whitney *U* test was used to compare non‐normally distributed continuous data. *p* < 0.05 was considered statistically significant.

Data processing and statistical analyses were performed using R version 4.4.1.

It was hypothesized that clinically relevant hypoxemic episodes would occur in 5% of participants during the day and 20% at night. With an *α* of 5% and a power of 80%, an estimated sample size of 150 participants was needed. Based on previous experience with continuous SpO_2_ monitoring, an additional sample size of 10% corresponding to 15 participants was added to account for potential data loss and measurement failures.

### 2.9 Use of Artificial Intelligence

During the preparation of this work, the authors used ChatGPT (OpenAI, April 2024 version) to assist with R programming and to improve the clarity and readability of the manuscript. After using this tool, the first author reviewed and validated all R code, and all authors reviewed and edited the manuscript as needed and take full responsibility for the content of the publication.

## 3 Results

### 3.1 Characteristics of Study Subjects

**FIGURE 1:**
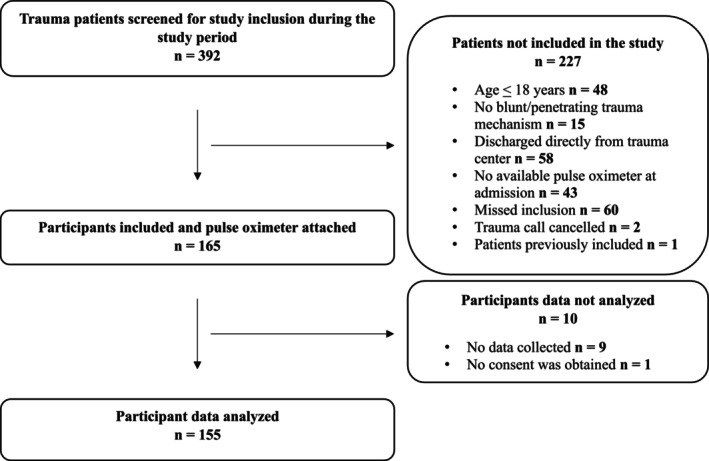
Patient inclusion flow. From February 20, 2024, to March 6, 2024, inclusion was limited to weekday day shifts (07:45–15:00). From March 7, 2024, to April 14, 2024, it was extended to 24 h coverage on weekdays. From April 15, 2024, to August 24, 2024, inclusion was further expanded to 24 h coverage 7 days a week.

From February 20, 2024, to August 24, 2024, 392 trauma patients were screened, 165 were included, and 155 (93.9%) were analyzed (Figure 1). From February 20, 2024, to March 6, 2024, inclusion was limited to weekday day shifts (07:45–15:00). From March 7, 2024, to April 14, 2024, it was extended to 24 h coverage on weekdays. From April 15, 2024, to August 24, 2024, inclusion was further expanded to 24 h coverage 7 days a week (Figure S1). The median age was 49 years (IQR 31–62), and 116 (74.8%) were male (Table 1). The median Injury Severity Score was 13 [9, 10, 11, 12, 13, 14, 15, 16, 17, 18, 19]. Almost all participants 146 (94.2%) received at least one potentially respiratory depressing drug from prehospital care through the first 24 h of hospitalization. Baseline characteristics of participants with only daytime or nighttime measurements are shown in Table S2. With one participant with nighttime measurements only, comparison would be uninformative.

**TABLE 1:** Baseline and clinical characteristics of study participants.

Characteristic	No. of participants with available data	Median (IQR) or *n* (%)
Age (years)	155	49 (31–62)
Sex, male	155	116 (74.8)
BMI (kg/m^2^)	126	25.1 (22.6–28.0)
Active smoker, participants	126	47 (37.3)
Lung disease, participants	155	7 (4.5)
Cardiovascular disease, participants	155	29 (18.7)
*Prehospital conditions*		
Dominant type of injury, participants	155	
Blunt		138 (89.0)
Penetrating		17 (11.0)
Mechanism of injury, participants	155	
Traffic		64 (41.2)
Falls		57 (36.8)
Hit by blunt object		18 (11.6)
Stabbing		12 (7.7)
Gunshot		4 (2.6)
Systolic blood pressure (mmHg)	139	131 (119–156)
Heart rate (bpm)	140	87 (76–103)
SpO_2_ (%)	138	96 (93–98)
Glasgow Coma Scale Score	134	15 (13–15)
Supplemental oxygen treatment, participants	145	86 (59.3)
Intubation, participants	155	29 (18.7)
Secondary transfer to Rigshospitalet, participants	155	22 (14.2)
*Trauma center conditions*		
Systolic blood pressure (mmHg)	150	134 (115–150)
Heart rate (bpm)	153	86 (71–100)
SpO_2_ (%)	155	98 (93–100)
Glasgow Coma Scale Score	147	15 (12–15)
Supplemental oxygen treatment, participants	144	98 (68.1)
Trauma center intubation, participants	155	9 (5.8)
Thoracic AIS score ≥ 2, participants^a^	135	53 (39.3)
Injury Severity Score	133	13 (9–19)
*Admission conditions*		
Potential respiratory depressing drugs in first 24 h, participants	155	146 (94.2)
ICU admission in first 24 h, participants	155	83 (53.5)
ICU—length of stay (days)^b^	80	1 (1–9)
Hospital—length of stay (days)^b^	150	7 (3–18)
Hospital discharge within first 24 h, participants^b^	150	13 (8.6)
30‐day mortality, participants	155	6 (3.9)
Supplemental oxygen treatment at different locations, participants		
Trauma center day	120	86 (71.7)
Trauma center night	51	24 (47.1)
Operating room day^c^	48	45 (93.8)
Operating room night^c^	27	23 (85.2)
Recovery room day	14	12 (85.7)
Recovery room night	15	9 (56.3)
ICU day	82	76 (92.7)
ICU night	77	67 (87.0)
Ward day	103	36 (35.0)
Ward night	90	28 (31.1)

*Note:* Arterial oxygen saturation by pulse oximetry (SpO_2_) was continuously monitored for 24 h in adult trauma patients to identify clinically relevant hypoxemic episodes. Data are presented as median (IQR) or *n* (%). *n* (%) indicates participants with the given characteristic, and percentage of participants with available data.

Abbreviations: AIS, Abbreviated Injury Scale; BMI, body mass index; bpm, beats per minute; ICU, intensive care unit; No., number; SpO_2_, arterial blood oxygen saturation measured by pulse oximetry.

^a^ AIS scores range from 0 to 6 and indicate the injury severity of traumatic lesions in different anatomical regions, with higher scores indicating higher severity.

^b^ Excludes participants who died in ICU or in‐hospital.

^c^ Some participants did not receive oxygen in the operating room when undergoing minor procedures, such as wound suturing under local anesthesia.

### 3.2 Main Results

Of the 155 included participants, 154 (99.4%) had at least one daytime measurement of SpO_2_, contributing a total of 1443.6 h of daytime data, while 136 (87.7%) had at least one nighttime measurement, contributing a total of 1429.3 h of nighttime data (Table 2). The primary outcome—clinically relevant hypoxemic episodes, defined as SpO_2_ < 90%, for > 5 min—was recorded 146 times in total: 73 episodes during daytime and 73 during nighttime. The IRs were 5.1 episodes per 100 participant‐hours during daytime and 5.1 episodes per 100 participant‐hours during nighttime, yielding an IRR of 1.01 (95% CI, 0.73–1.4; *p* = 0.95). Daytime episodes occurred among 34 (22.1%) participants with daytime measurements, while nighttime episodes occurred among 24 (17.6%) participants with nighttime measurements (Table 2).

**TABLE 2:** SpO_2_ measurement and hypoxemia in the first 24 h after trauma.

	Daytime 08:00–19:59	Nighttime 20:00–07:59	Incidence rate ratio (95% CI)	*p*
Duration of SpO_2_ measurement (h)	1443.6	1429.3	**—**	**—**
Participants with measurement (% of participants included in study)	154 (99.4%)	136 (87.7%)	**—**	**—**
Duration of SpO_2_ measurement per participant (h), median (IQR)	10.9 (7.6–11.8)	11.9 (7.5–12.0)	**—**	**—**
Hypoxemic episodes^a^	73	73	**—**	**—**
Incidence rate of hypoxemic episodes,^a^ per 100 participant‐hours	5.1	5.1	1.01^b^ (0.73–1.4)^c^	0.95^c^
Participants with hypoxemic episodes^a^ (% of participants with period monitoring)	34 (22.1%)	24 (17.6%)	**—**	**—**
Hypoxemic episodes^a^ per participant with at least one episode, median (IQR)	1 (1–3)	2 (1–2)	**—**	**—**
Prolonged hypoxemic episodes^d^	13	14	**—**	**—**
Incidence rate of prolonged hypoxemic episodes^d^, per 100 participant‐hours	0.9	1.0	1.09^b^ (0.50–2.31)^c^	0.83^c^
Participants with prolonged hypoxemic episodes^d^	7 (4.5%)	8 (5.9%)	**—**	**—**
Prolonged hypoxemic episodes^d^ per participant with at least one episode, median (IQR)	1 (1–2)	1 (1–1)	**—**	**—**
Cumulative duration of SpO_2_ < 90% (h)	45.5	50.0	—	**—**
Cumulative duration of SpO_2_ < 90% per participant, minutes, median (IQR)	5.5 (0.7–19.4)	3.0 (0.2–12.7)		0.05^e^
Intensive care unit incidence rate of hypoxemic episodes^a^, per 100 participant‐hours	3.3	2.4	0.72^b^ (0.38–1.37)^c^	0.31^c^
Ward incidence rate of hypoxemic episodes^a^, per 100 participant**—**hours	6.4	8.5	1.33^b^ (0.88–2.01)^c^	0.18^c^
Trauma center incidence rate of hypoxemic episodes^a^, per 100 participant‐hours	6.7	4.5	0.67^f^ (0.01–5.22)^f^	1^f^
Operating room incidence rate of hypoxemic episodes^a^, per 100 participant‐hours	6.6	0	**—**	**—**
Recovery room incidence rate of hypoxemic episodes^a^, per 100 participant‐hours	0	0	**—**	**—**

*Note:* Arterial oxygen saturation by pulse oximetry (SpO_2_) was continuously monitored for 24 h in adult trauma patients to identify clinically relevant hypoxemic episodes.

^a^ SpO_2_ < 90% for > 5 min.

^b^ Poisson regression.

^c^ Wald method.

^d^ SpO_2_ < 90% for > 30 min.

^e^ Mann–Whitney *U* test.

^f^ Exact Poisson test.

### 3.3 Secondary Outcomes

**FIGURE 2:**
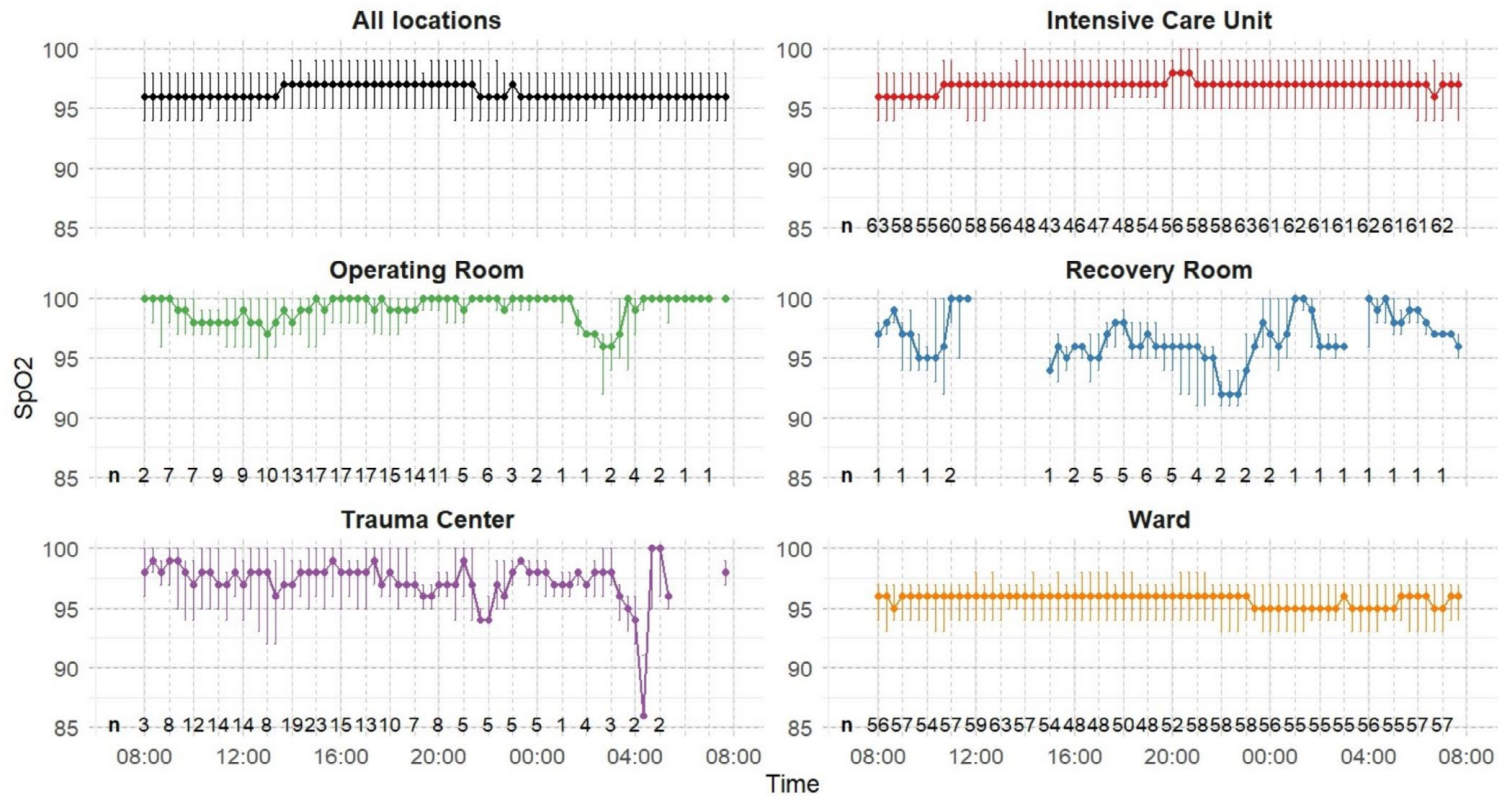
SpO_2_ 20‐min medians with interquartile ranges the first day after hospital admission after trauma, stratified by hospital location. Arterial oxygen saturation by pulse oximetry (SpO_2_) was continuously monitored for 24 h in adult trauma patients to identify clinically relevant hypoxemic episodes. *n*: Number of participants included in the calculation of the median at each hourly interval.

Prolonged hypoxemic episodes, defined as SpO_2_ < 90%, for > 30 min, were recorded 27 times in total: 13 episodes during daytime and 14 during nighttime. The IRs were 0.9 and 1.0 episodes per 100 participant‐hours during daytime and nighttime respectively, yielding an IRR of 1.09 (95% CI, 0.50–2.31; *p* = 0.83). Prolonged daytime episodes occurred in 7 (4.5%) participants with daytime measurements, and nighttime episodes occurred in 8 (5.9%) participants with nighttime measurements. The cumulative duration of SpO_2_ < 90% per participant showed a median of 5.5 min (0.7–19.4) during daytime and 3.0 min (0.2–12.7) during nighttime (*p* = 0.05). In total, 45.5 h (3.2%) of recorded daytime measurement and 50.0 h (3.5%) of recorded nighttime measurement showed an SpO_2_ < 90% (Table S3). There was considerable variation in the number of participants admitted across different hospital locations (Table S4) and hence in measured time across locations during both daytime and nighttime, ranging from 18.4 h of cumulated nighttime measurement in the recovery room to 711.9 h of cumulated nighttime measurement in the ICU (Table S3). The IR of clinically relevant hypoxemic episodes in the ICU were 3.3 per 100 participant‐hours during daytime and 2.4 during nighttime, yielding an IRR of 0.72 (95% CI, 0.38–1.37; *p* = 0.31). In the trauma center the IRs were 6.7 during daytime and 4.5 during nighttime, yielding an IRR of 0.67 (95% CI, 0.01–5.22; *p* = 1). The operating room had a daytime IR of 6.6, but no episodes were recorded during nighttime, nor were any recorded in the recovery room. In the ward, the IRs were 6.4 during daytime and 8.5 during nighttime, resulting in an IRR of 1.33 (95% CI, 0.88–2.01; *p* = 0.18). A 24 h comparison of median SpO_2_ levels across locations is shown in Figure 2.

## 4 Discussion

In this study, the IRs of clinically relevant hypoxemic episodes—defined as SpO_2_ < 90%, for > 5 min—were 5.1 per 100 participant‐hours during both daytime and nighttime within the first 24 h following admission to the trauma center, indicating no difference between the two periods. Similarly, no differences were found in the IRs of prolonged hypoxemic episodes, the cumulative duration of SpO_2_ < 90% per participant, or across any hospital locations. The highest IR of hypoxemic episodes was recorded in the ward at night. Furthermore, it was the only location with a higher nighttime IR compared to daytime, though this difference was not statistically significant.

Given that nearly all participants received at least one potentially respiratory depressing drug during the early treatment phase, this routine clinical practice justifies focusing on hypoxemia in all trauma patients, regardless of injury pattern.

Unlike previous studies [20, 21, 22], the present data showed no difference in hypoxemia occurrence between daytime and nighttime. This discrepancy may be explained by variation in patient populations, outcome measures, and the level of monitoring provided as standard care.

The detection of in‐hospital hypoxemic episodes in this study aligns with findings from both the recent multicenter randomized controlled TRAUMOX2 trial, which compared restrictive and liberal supplemental oxygen strategies shortly after trauma for 8 h in 1508 adult trauma patients [9, 10], and previous prehospital studies reporting hypoxemia incidences between 28.7% and 38.4% [6, 7, 8]. However, several key differences between the present and the previous studies limit direct comparability. These include variations in measurement duration and frequency, outcome definitions, and statistical methodologies. Additionally, the TRAUMOX2 trial involved a supplemental oxygen‐related intervention, which may also have increased clinical attention to oxygen saturation levels among trial participants, resulting in conditions that do not necessarily reflect standard care.

### 4.1 Limitations

First, the median monitoring time per participant was 1 h longer at nighttime compared to daytime, and 18 additional participants had recorded measurements during daytime than at nighttime. These discrepancies may be partly explained by hospital discharges and transfers preferably occurring during daytime hours. Some participants admitted during the day were either discharged or transferred to a different hospital during the same day, thereby completely omitting data the following nighttime and resulting in incomplete daytime measuring. This could introduce attrition bias, as only the least injured participants are likely to be discharged before completing the full 24 h of monitoring. As a result, potential episode‐free hours may be removed. Furthermore, the investigators were mainly present at the study site during daytime. They would directly contact the departments where participants were admitted to ensure that the project equipment was functioning correctly. At night, the monitoring quality checks were performed remotely by phone and less frequently than in daytime. On some occasions, investigators went to visit departments the following morning only to find that the equipment had not been recording SpO_2_ data overnight. Besides early discharges and transfers, the shorter median monitoring time per participant in daytime may be explained by more diagnostic tests and procedures performed during the day, increasing the risk of research monitoring equipment being removed while the participant underwent, for example, additional magnetic resonance imaging (MRI) scans, as the equipment had to be taken off before the scan. This could lead to interruptions or early termination of monitoring if the equipment was not promptly reattached. Second, the study did not collect data on skin pigmentation, which has previously been shown to potentially affect the accuracy of pulse oximetry by overestimating SpO_2_ levels in patients with darker skin tones [25, 26]. If a significant proportion of the included participants had a darker skin pigmentation, the true occurrence of hypoxemia may have been underestimated. Third, supplemental oxygen treatment was not characterized in detail, limiting the ability to evaluate a potential association between the specifics of supplemental oxygen treatment and the occurrence or severity of hypoxemia. Fourth, the study's observational design inherently introduces confounding by indication. The most severely injured participants were admitted to the ICU or operating room where monitoring is more consistent, which theoretically could prevent desaturations. Fifth, the study was powered to detect relatively large differences in hypoxemia IRs between daytime and nighttime, and the observed CIs were wide, indicating limited precision to rule out smaller differences.

### 4.2 Strengths

The present study has some strengths. First, the included trauma population reflects the general characteristics of adult trauma patients reported in other studies, supporting the applicability of the findings to this group [1, 2, 9]. It is important to note that the present study included all participants across various hospital locations, not limited to those classified as critically injured. Second, SpO_2_ was measured using standardized equipment across all participants, enhancing the study's reproducibility. Third, the study reflects hypoxemia under standard treatment conditions, with no additional supplemental oxygen interventions provided and pulse oximeter alarm thresholds set restrictively. This enhances the clinical relevance of the findings, as they reflect real‐world practice under current guidelines.

### 4.3 Meaning and Potential Implications of Findings

The detection of clinically relevant hypoxemic episodes in this study aligns with previous findings from both prehospital and in‐hospital studies, emphasizing the continued need for heightened awareness of hypoxemia risk among trauma patients in the initial period following injury. Increased vigilance may be particularly important in the ward, especially at night, where the highest IR of hypoxemic episodes was found. It is well established that monitoring levels in the ward are lower than in other hospital locations [27]. We advocate for a continued focus on hypoxemia among trauma patients. This study identifies a potential issue in ward settings that requires further investigation before management recommendations can be formulated.

## 5 Conclusion

On average, 5.1 clinically relevant hypoxemic episodes occurred per 100 participant‐hours of continuous SpO_2_ monitoring during the first 24 h of hospitalization following trauma. The study found similar IRs of clinically relevant hypoxemic episodes at daytime and nighttime.

## Author Contributions

**Jacob Jensen‐Abbew:** conceptualization, data curation, formal analysis, funding acquisition, investigation, methodology, project administration, resources, validation, visualization, writing – original draft. **Emma Atsuko Tsuchiya:** data curation, investigation, project administration, validation, writing – review and editing. **Tobias Arleth:** conceptualization, investigation, methodology, writing – review and editing. **Felicia Dinesen:** conceptualization, investigation, methodology, writing – review and editing. **Carl Johan Queitsch:** conceptualization, methodology, writing – review and editing. **Martin Von Magius:** investigation, resources, writing – review and editing. **Michelle Icka Christensen:** investigation, writing – review and editing. **Karl Peter Damgård Madsen:** investigation, writing – review and editing. **Oscar Rosenkrantz:** conceptualization, formal analysis, investigation, methodology, validation, writing – review and editing. **Jacob Steinmetz:** conceptualization, formal analysis, funding acquisition, methodology, project administration, resources, supervision, validation, writing – review and editing.

## Funding

The work was supported by The Danish Medical Association's Research Fund (grant 2023‐0043). The Danish Medical Association's Research Fund had no role in the study design, data collection, data analysis or interpretation, manuscript preparation, or the decision to submit the article for publication.

## Ethics Statement

The study was approved (H‐23065206) on January 3, 2024, with a supplemental approval (110381) on April 4, 2024, by the Regional Research Ethics Committee in the Capital Region, and approved by the Capital Region of Denmark Data Controller's Register (Privacy: P‐2023‐15008).

## Consent

Informed consent was obtained for all participants in accordance with the Danish Medical Research Ethics Committee's emergency research procedure.

## Conflicts of Interest

The authors declare no conflicts of interest.

## Supporting information


**Data S1:** aas70220‐sup‐0001‐Supinfo.docx.

## Data Availability

A deidentified dataset will be made available upon reasonable request and relevant permissions, by contacting Jacob Jensen‐Abbew, MD, at jacob.jensen-abbew.01@regionh.dk or Professor Jacob Steinmetz, MD, PhD, at jacob.steinmetz@regionh.dk.
