# Supplementary material for: Occurrence of Hypoxemia the First Day After Trauma Assessed by Continuous Pulse Oximetry
Source: Acta Anaesthesiol Scand. 2026 Mar 16;70(4):e70220. doi: 10.1111/aas.70220 (PMC12992667; doi:10.1111/aas.70220)

**Supplementary**

**Article title:** *Occurrence of Hypoxemia the First Day After Trauma Assessed by Continuous Pulse Oximetry*

**Journal:** *Acta Anaesthesiologica Scandinavica*

**Correspondence:** Jacob Jensen-Abbew, MD, Department of Anaesthesiology, Centre of Head and Orthopaedics, Copenhagen University Hospital – Rigshospitalet

Inge Lehmanns Vej 6, Section 6011, DK-2100 Copenhagen, Denmark

E-mail: jacob.jensen-abbew.01@regionh.dk

Phone: +45 28 73 35 22

**Content**

[TABLES 2](#_Toc221182549)

[Table S1 Drugs classified as potentially respiratory depressing 2](#_Toc221182550)

[Table S2 Baseline and clinical characteristics: participants with daytime-only (n=19) and nighttime-only (n=1) measurements 3](#_Toc221182551)

[Table S3 SpO_2_ measuring time, cumulative hypoxemia duration and hypoxemic episodes by location 5](#_Toc221182552)

[Table S4 Number of participants by hospital location during the first 24 hours of admission 5](#_Toc221182553)

[FIGURES 6](#_Toc221182554)

[Figure S1 Timeline of inclusion hours 6](#_Toc221182555)

[PROTOCOL 7](#_Toc221182556)

# TABLES

## Table S1 Drugs classified as potentially respiratory depressing

| Drug class | Drug group | Drugs |
| --- | --- | --- |
| **Opioids** | - | Morphine, Fentanyl, Oxycodone, Alfentanil, Remifentanil, Tramadol |
| **Sedatives** | Benzodiazepines | Midazolam, Diazepam, Lorazepam, Alprazolam, Clonazepam, Chlordiazepoxide, Bromazepam |
|  | Non-benzodiazepine hypnotics | Zopiclone, Zolpidem, Eszopiclone |
|  | Barbiturates | Phenobarbital, Thiopental, Secobarbital, Pentobarbital |
|  | Sedating Antihistamines | Diphenhydramine, Hydroxyzine, Promethazine |
|  | General Anesthetics | Sevoflurane, Isoflurane, Desflurane, Propofol, Ketamine |
|  | Gabapentinoids | Gabapentin, Pregabalin |
|  | Antipsychotics | Clozapine, Olanzapine, Haloperidol, Quetiapine, Droperidol |
|  | Other sedatives | Clonidine |
| **Other** | Tricyclic Antidepressants | Amitriptyline, Nortriptyline, Imipramine |
|  | Muscle Relaxants | Baclofen, Tizanidine, Cyclobenzaprine, Methocarbamol, Cisatracurium, Rocuronium bromide, Suxamethonium |
|  | Local Anesthetics | Lidocaine, Bupivacaine, Ropivacaine |
|  | Anticholinergic Drugs | Atropine, Scopolamine |

*Arterial oxygen saturation by pulse oximetry (SpO_2_) was continuously monitored for 24 hours in adult*
*trauma patients to identify clinically relevant hypoxemic episodes.*
*Drugs classified as potentially respiratory depressing based on their pharmacological effects and documented side effects*

## Table S2 Baseline and clinical characteristics: participants with daytime-only (n=19) and nighttime-only (n=1) measurements

|  | **Only daytime measurement** | | **Only nighttime measurement** | |
| --- | --- | --- | --- | --- |
| **Characteristic** | No. of Participants with available data | Median (IQR) or n (%) | No. of Participants with available data | Median (IQR) or n (%) |
| Age, years | 19 | 40 (31–65) | 1 | 82 (-) |
| Sex, male | 19 | 15 (78.9) | 1 | 1 (100) |
| BMI, kg/m^2^ | 16 | 24.9 (23.7–26.4) | 0 | - |
| Active smoker, participants | 17 | 4 (23.5) | 1 | 1 (100) |
| Lung disease, participants | 19 | 0 (0) | 1 | 0 (0) |
| Cardiovascular disease, participants | 19 | 4 (21.1) | 1 | 0 (0) |
| **Prehospital conditions** |  |  |  |  |
| Dominant type of injury, participants | 19 |  | 1 |  |
| *Blunt* |  | 16 (84.2) |  | 1 (100) |
| *Penetrating* | | 3 (15.8) |  | - |
| Mechanism of injury, participants | 19 |  | 1 |  |
| *Traffic* |  | 7 (36.8) |  | - |
| *Falls* |  | 9 (47.3) |  | 1 (100) |
| *Hit by blunt object* | | 0 (0) |  | - |
| *Stabbing* | | 3 (15.8) |  | - |
| *Gunshot* | | 0 (0) |  | - |
| Systolic Blood Pressure, mm Hg | 17 | 129 (120–151) | 1 | 155 (-) |
| Heart Rate, bpm | 18 | 89 (77–95) | 1 | 66 (-) |
| SpO_2_, % | 17 | 95 (92–98) | 1 | 97 (-) |
| Glasgow Coma Scale Score | 18 | 14 (10–15) | 1 | 14 (-) |
| Supplemental oxygen treatment, participants | 17 | 10 (58.8) | 1 | 1 (100) |
| Intubation, participants | 19 | 6 (31.6) | 1 | 0 (0) |
| Secondary transfer to Rigshospitalet, participants | 19 | 1 (5.3) | 1 | 0 (0) |
| **Trauma center conditions** |  |  |  |  |
| Systolic Blood Pressure, mm Hg | 17 | 127 (114–144) | 1 | 147 (-) |
| Heart Rate, bpm | 18 | 91 (79–105) | 1 | 74 (-) |
| SpO_2_, % | 19 | 99 (94–100) | 1 | 100 (-) |
| Glasgow Coma Scale Score | 18 | 15 (3–15) | 1 | 14 (-) |
| Supplemental oxygen treatment, participants | 18 | 11 (61.1) | 1 | 1 (100) |
| Trauma center intubation, participants | 19 | 1 (5.3) | 1 | 0 (0) |
| Thoracic AIS > 2, participants^a^ | 17 | 5 (29.4) | 1 | 0 (0) |
| Injury Severity Score | 15 | 10 (4–16) | 1 | 1 (-) |
| **Admission conditions** |  |  |  |  |
| Potential respiratory depressing drugs in first 24 hours, participants | 19 | 17 (89.4) | 1 | 0 (0) |
| ICU admission in first 24 hours, participants | 19 | 9 (47.3) | 1 | 0 (0) |
| ICU - Length of Stay, days^b^ | 7 | 1 (0–13) | - | - |
| Hospital - Length of Stay, days^b^ | 17 | 4 (1–7) | 1 | 1 (-) |
| Hospital discharge within first 24 hours, participants^b^ | 17 | 3 (17.6) | 1 | 1 (100) |
| 30-day mortality, participants | 19 | 2 (10.5) | 1 | 0 (0) |
| Supplemental oxygen treatment at different locations, participants |  |  |  |  |
| *Trauma center day* | 15 | 11 (73.3) | 1 | 1 (100) |
| *Trauma center night* | 6 | 2 (33.3) | 1 | 1 (100) |
| *Operating room day^c^* | 3 | 3 (100) | - | - |
| *Operating room night^c^* | 3 | 3 (100) | - | - |
| *Recovery room day* | 1 | 1 (100) | - | - |
| *Recovery room night* | 2 | 0 (0.0) | - | - |
| *ICU day* | 9 | 9 (100) | - | - |
| *ICU night* | 4 | 4 (100) | - | - |
| *Ward day* | 12 | 3 (25.0) | 1 | 0 (0) |
| *Ward night* | 10 | 2 (20.0) | 1 | 1 (100) |

*Notes: Arterial oxygen saturation by pulse oximetry (SpO_2_) was continuously monitored for 24 hours in adult trauma patients to identify clinically relevant hypoxemic episodes.*
*Data are presented as median (IQR) or n (%).*
*n (%) indicates participants with the given characteristic, and percentage of participants with available data.*
*Abbreviations: No.: Number; BMI: Body Mass Index; bpm: Beats per minute; SpO_2_: Arterial blood oxygen saturation measured by pulse oximetry; AIS: Abbreviated Injury Scale; ICU: Intensive Care Unit*

^a^: *AIS scores range from 0 to 6 and indicate the injury severity of traumatic lesions in different anatomical regions with higher scores indicating higher severity*
^b^*: Excludes participants who died in ICU or in-hospital*
*^c^:* *Some participants did not receive oxygen in the operating room when undergoing minor procedures, such as wound suturing under local anesthesia*

## Table S3 SpO_2_ measuring time, cumulative hypoxemia duration and hypoxemic episodes by location

|  | | **Daytime (08.00**–**19.59)** | **Nighttime**  **(20.00**–**07.59)** | | |  |
| --- | --- | --- | --- | --- | --- | --- |
| **Cumulative duration of SpO_2_ < 90%/Cumulative time of measurement, hours, n(%)** | | | | | | |
| *Total* | 45.5/1,443.6 = 3.2% | | | 50.0/1,429.3 = 3.5% |  | |
| *Intensive Care Unit* | 13.8/600.7 = 2.3% | | | 12.1/711.9 = 1.7% |  | |
| *Ward* | 23.4/595.2 = 3.9% | | | 36.5/647.1 = 5.6% |  | |
| *Trauma Center* | 5.0/104.7 = 4.8% | | | 0.6/22.3 = 2.7% |  | |
| *Operating Room* | 3.0/121.2 = 2.5% | | | 0.2/29.6 = 0.7% |  | |
| *Recovery Room* | 0.4/21.8 = 1.8% | | | 0.6/18.4 = 3.3% |  | |
| **Time of measurement per patient by location, hours, median (IQR)** | | | | | | |
| *Intensive Care Unit* | 8.2 (5.7–10.4) | | | 11.9 (9.8–12.0) |  | |
| *Ward* | 6.2 (2.9–9.0) | | | 11.1 (5.4–11.9) |  | |
| *Trauma Center* | 0.9 (0.4–1.3) | | | 0.6 (0.3–1.0) |  | |
| *Operating Room* | 3.0 (2.0–3.6) | | | 1.3 (0.6–2.0) |  | |
| *Recovery Room* | 1.8 (1.2–2.3) | | | 1.5 (1.3–2.2) |  | |
| **Episodes of hypoxemia^a^** | | | | | | |
| *Intensive Care Unit* | 20 | | | 17 |  | |
| *Ward* | 38 | | | 55 |  | |
| *Trauma Center* | 7 | | | 1 |  | |
| *Operating Room* | 8 | | | 0 |  | |
| *Recovery Room* | 0 | | | 0 |  | |

*Notes: Arterial oxygen saturation by pulse oximetry (SpO_2_) was continuously monitored for 24 hours in adult trauma patients to identify clinically relevant hypoxemic episodes.*^a^*: SpO_2_ < 90%, for > 5 minutes*

## Table S4 Number of participants by hospital location during the first 24 hours of admission

| **Location** | **n (%)** |
| --- | --- |
| *Intensive Care Unit* | 83 (53.5) |
| *Ward* | 104 (67.1) |
| *Trauma Center* | 155 (100.0) |
| *Operating Room* | 59 (38.1) |
| *Recovery Room* | 21 (13.5) |

*Notes: Arterial oxygen saturation by pulse oximetry (SpO_2_) was continuously monitored for 24 hours in adult trauma patients to identify clinically relevant hypoxemic episodes.
All participants were initially admitted to the trauma center and may have been in multiple other hospital locations during the first 24 hours.
n(%): Number and percentage of participants (N = 155) in each location*

# FIGURES

**February 20, 2024 – March 6, 2024**

**(5 participants included)**

**March 7, 2024 – April 14, 2024**

**(19 participants included)**

**April 15, 2024 – August 24, 2024**

**(141 participants included)**

## Figure S1 Timeline of inclusion hours

*Notes: The inclusion hours were extended over the course of the study (as shown above), and the corresponding number of participants included in each period is presented*


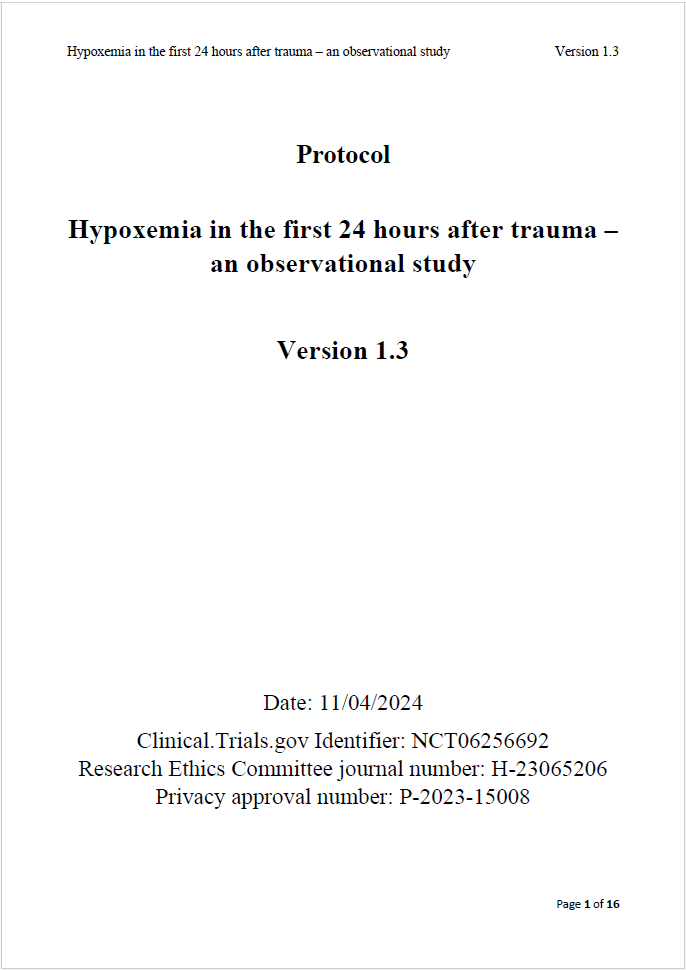


# PROTOCOL


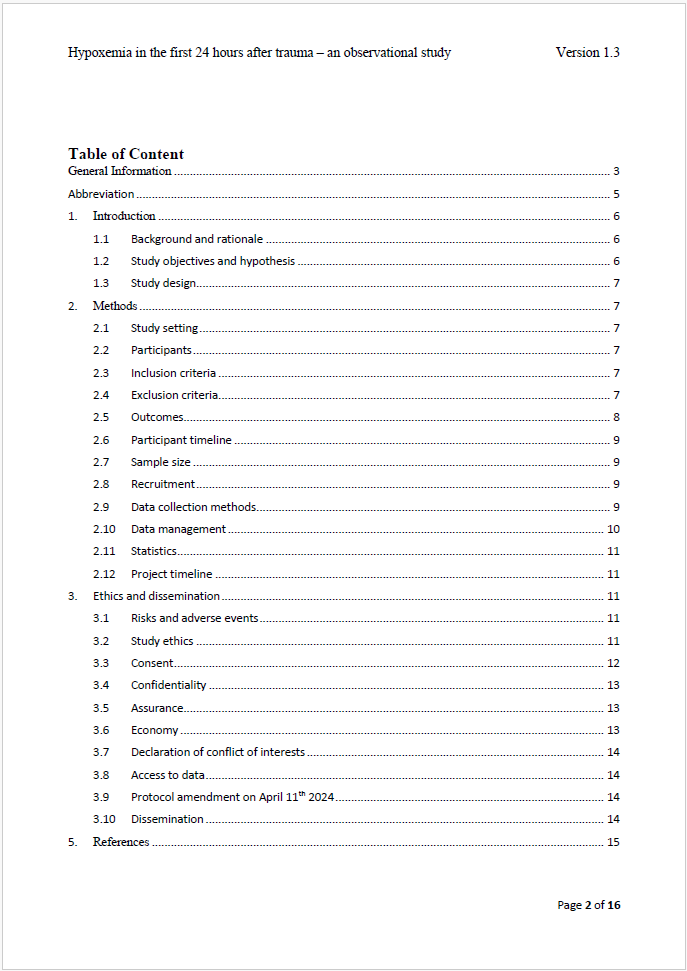


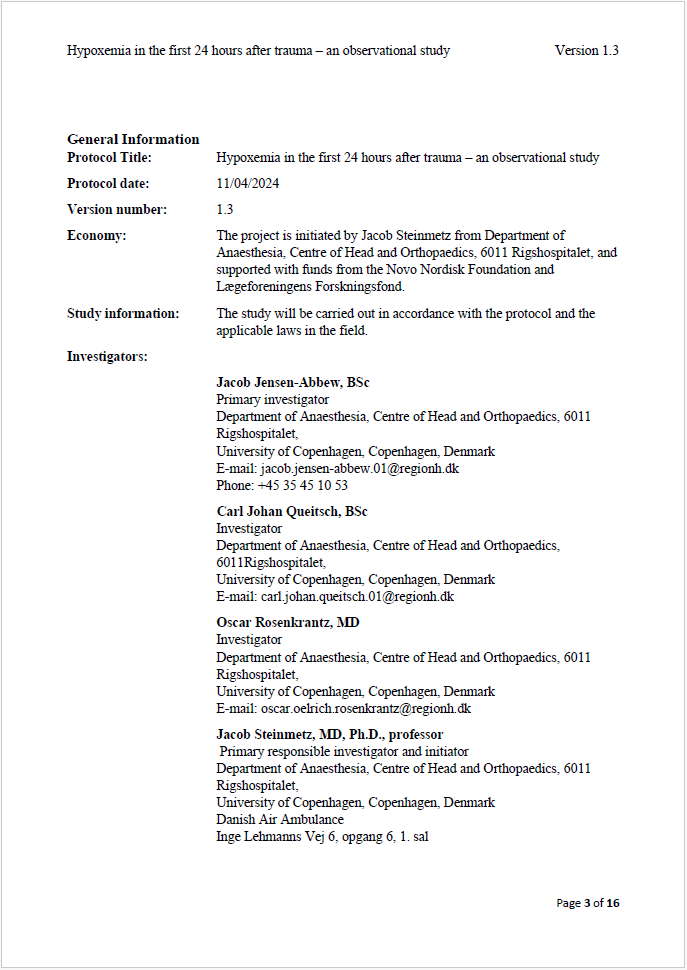

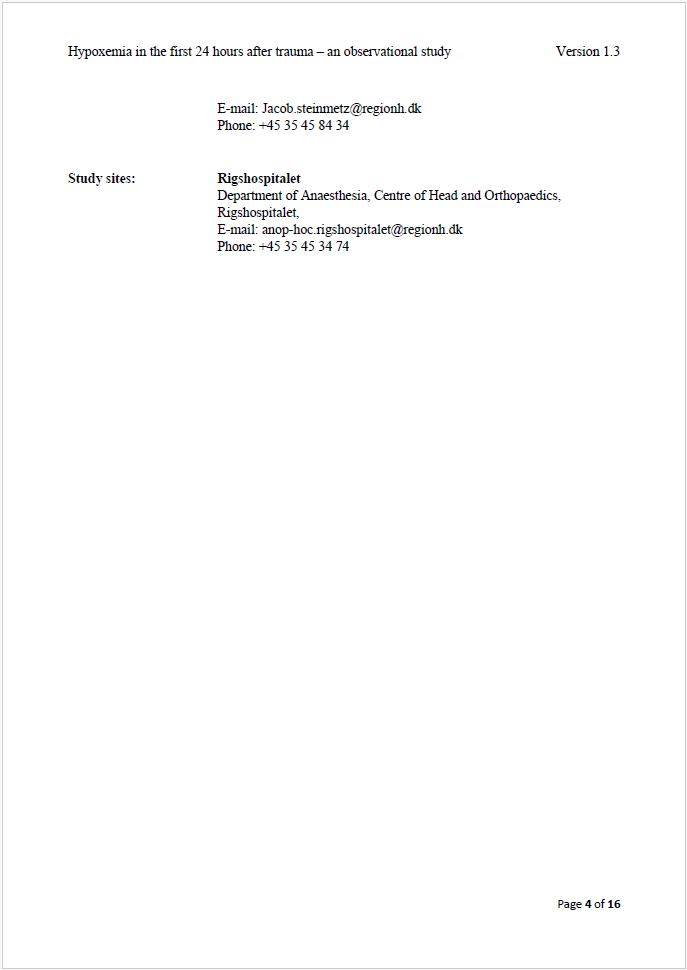

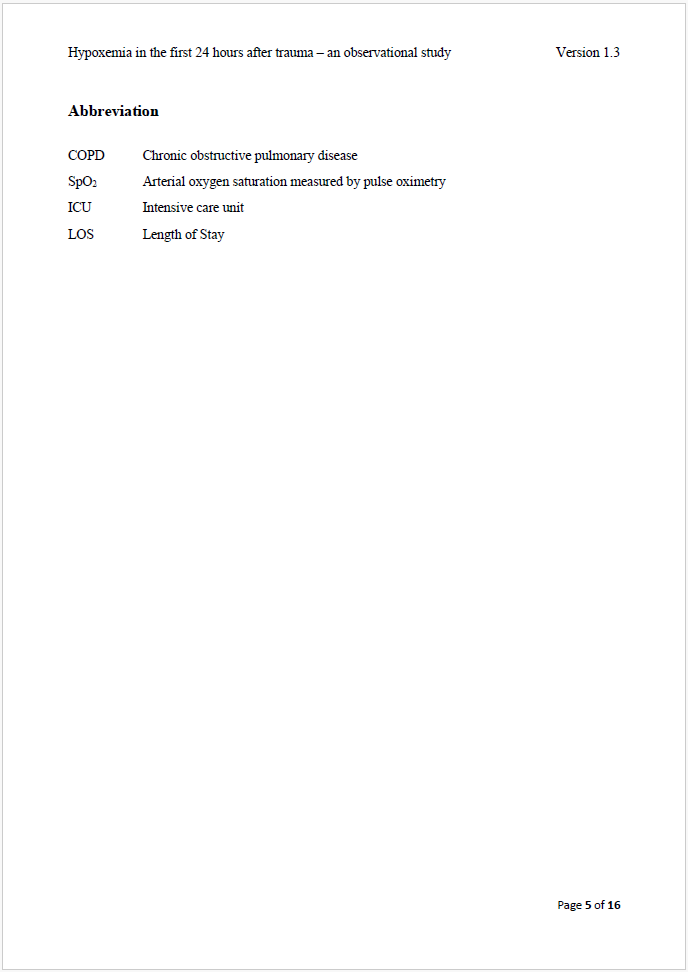

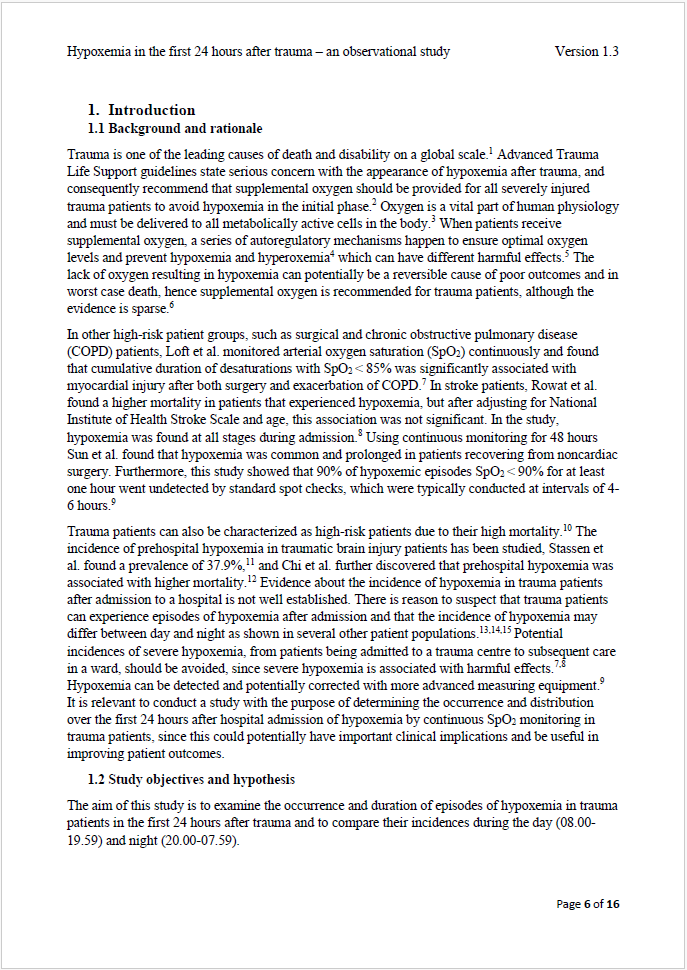

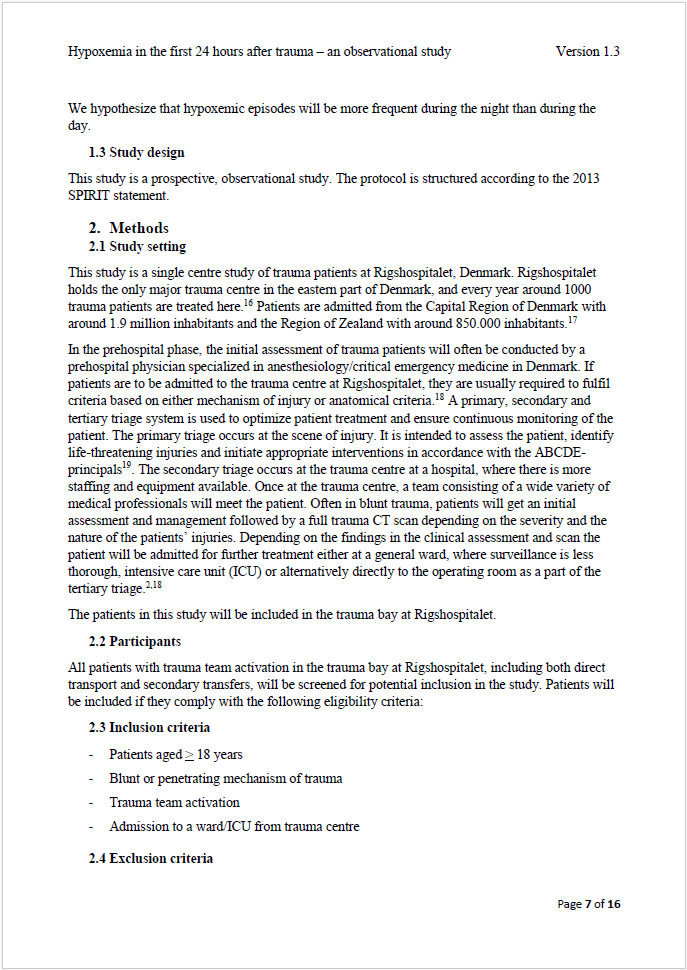

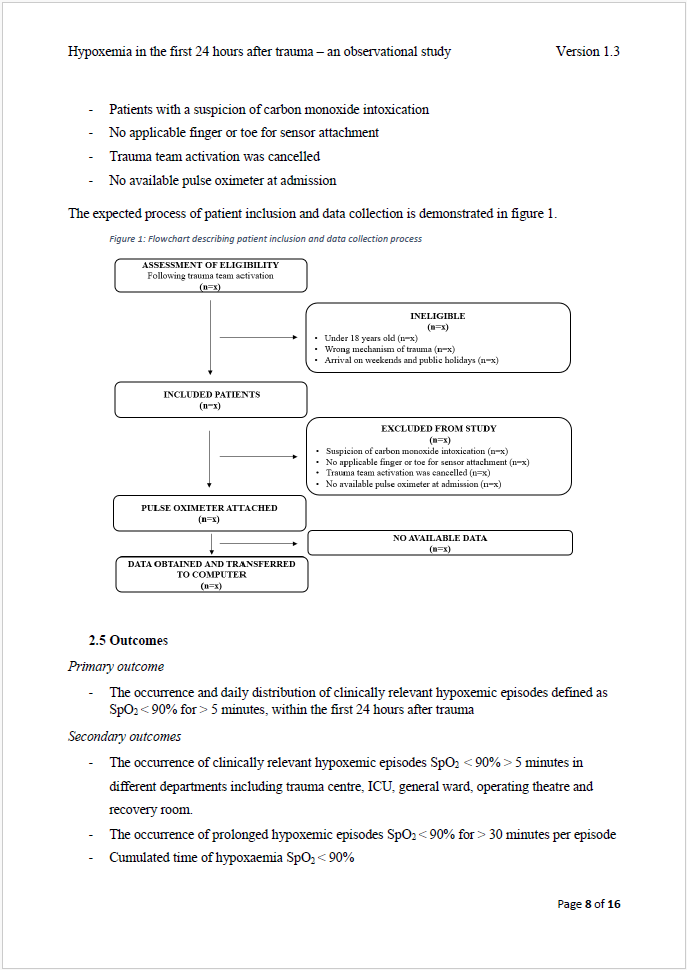

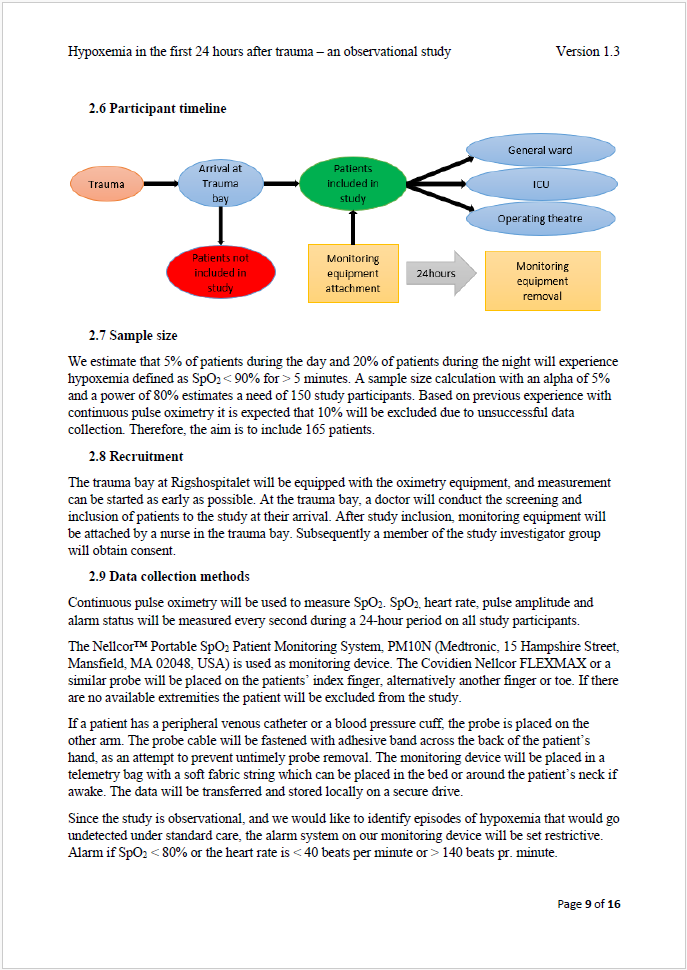

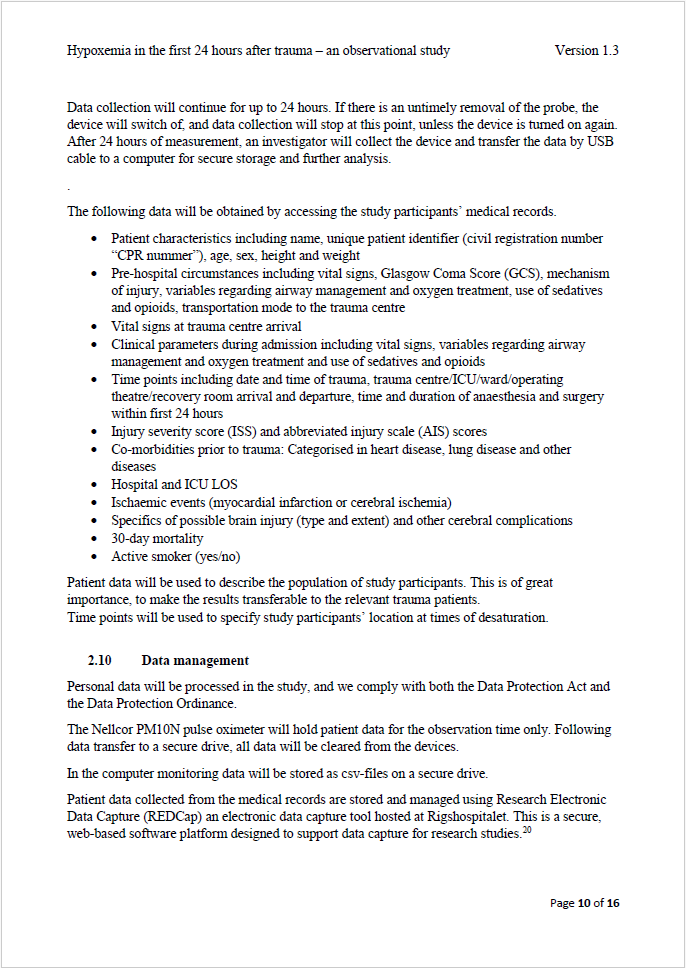

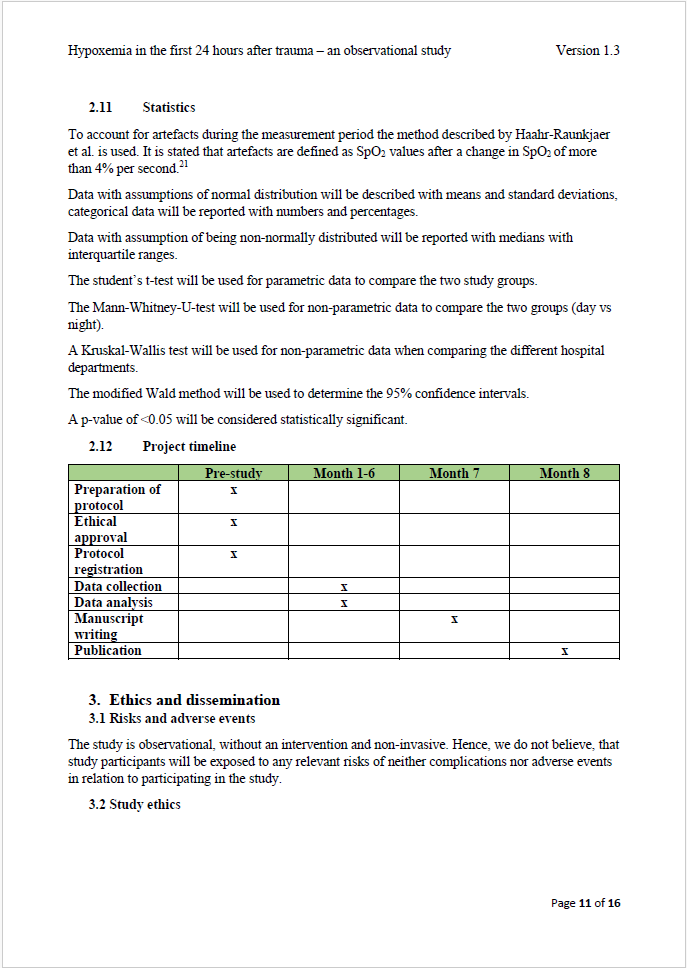

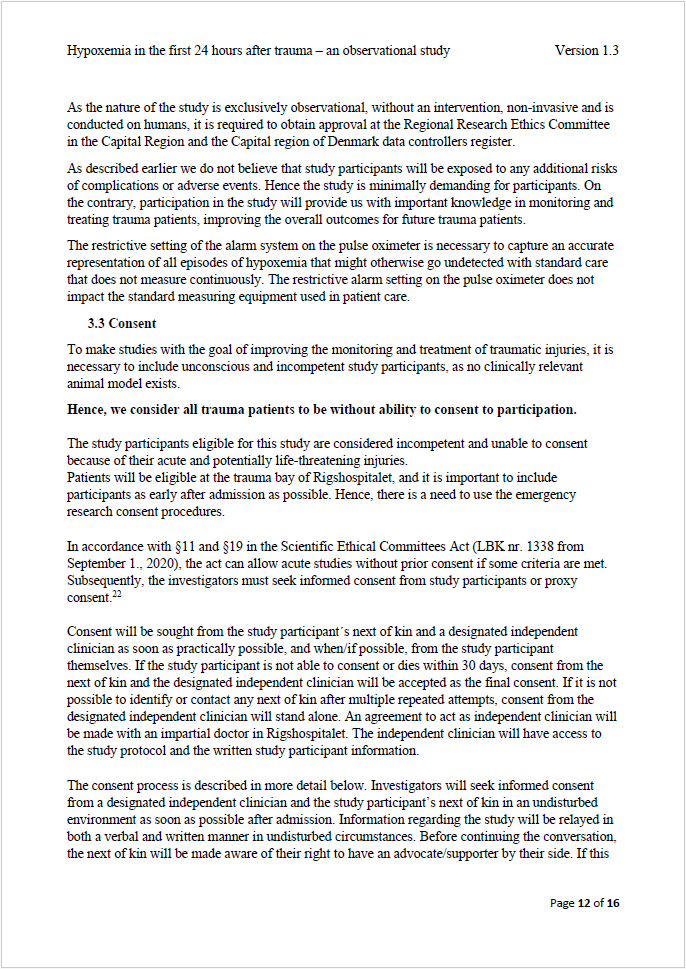

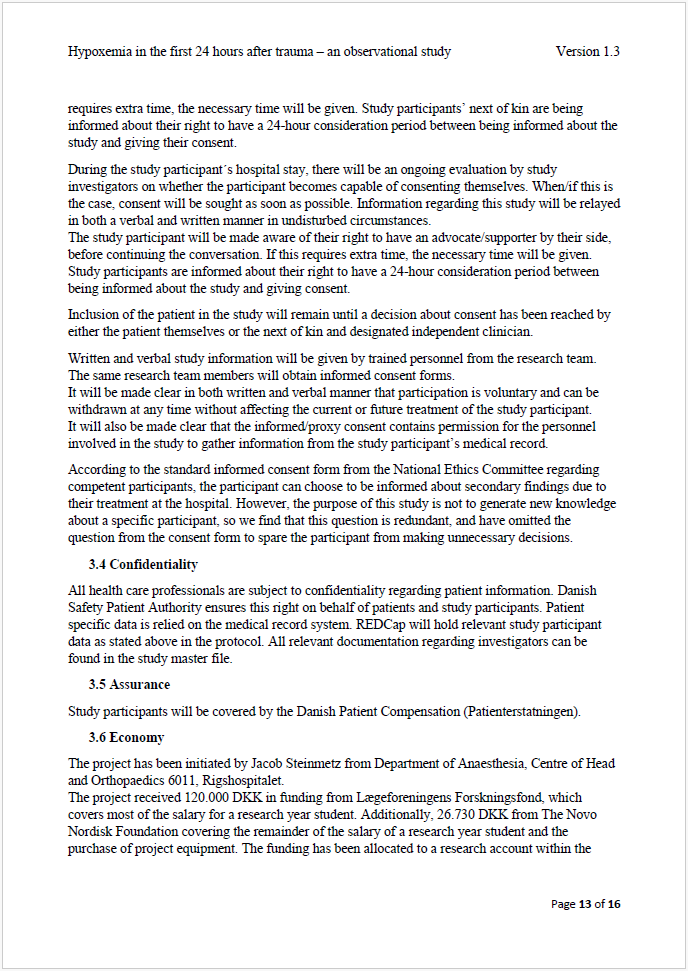

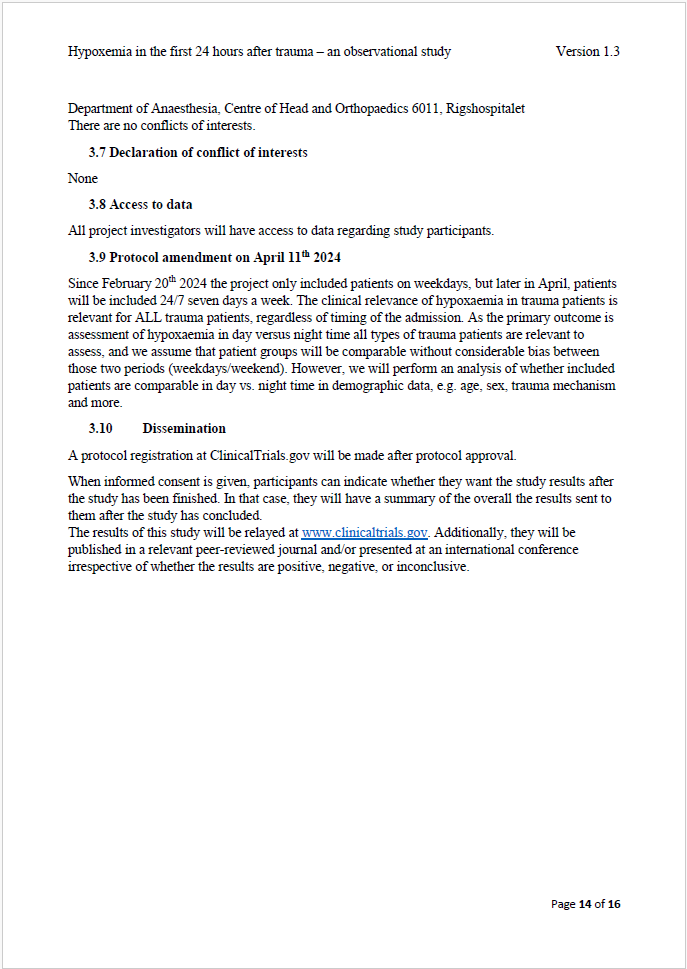

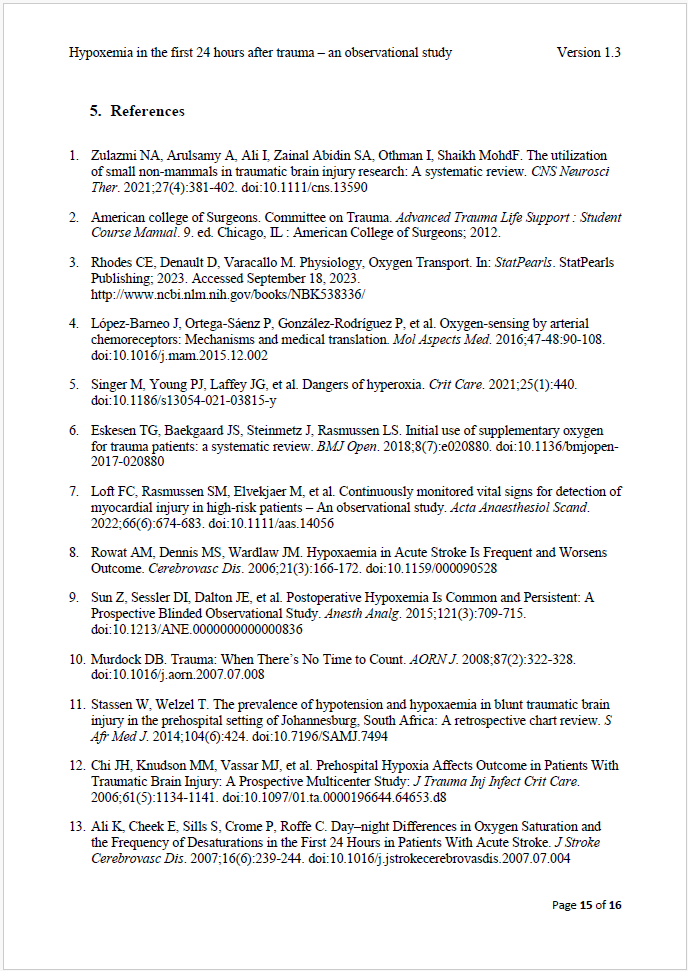

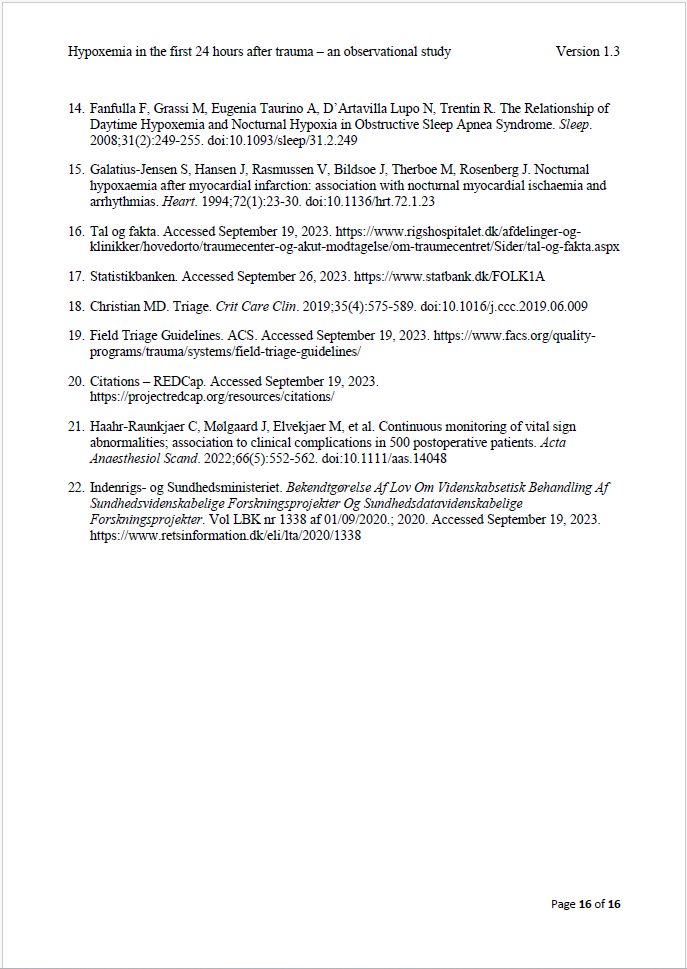

Supplement: Supplementary file 1 — Data S1: aas70220‐sup‐0001‐Supinfo.docx. [file AAS-70-0-s001.docx]
